# Anti-MOG antibody-positive ADEM following infectious mononucleosis due to a primary EBV infection: a case report

**DOI:** 10.1186/s12883-017-0858-6

**Published:** 2017-04-19

**Authors:** Yoshitsugu Nakamura, Hideto Nakajima, Hiroki Tani, Takafumi Hosokawa, Shimon Ishida, Fumiharu Kimura, Kimihiko Kaneko, Toshiyuki Takahashi, Ichiro Nakashima

**Affiliations:** 1 Division of Neurology, Department of Internal Medicine IV, Osaka Medical College, Daigakumachi 2-7, Takatsukishi, Osaka, 569-8686 Japan; 2 Division of Neurology, Tohoku University School of Medicine, Seiryomachi 1-1, Aobaku, Sendai, 980-8574 Japan; 3 Division of Neurology, NHO Yonezawa Hospital, Ozimisawa 26100-1, Yonezawashi, Yamagata, 992-1202 Japan

**Keywords:** Myelin oligodendrocyte glycoprotein, Acute disseminate encephalomyelitis, Epstein–Barr virus, Transverse myelitis, Antecedent infection, Case report

## Abstract

**Background:**

Anti-Myelin oligodendrocyte glycoprotein (MOG) antibodies are detected in various demyelinating diseases, such as pediatric acute disseminated encephalomyelitis (ADEM), recurrent optic neuritis, and aquaporin-4 antibody-seronegative neuromyelitis optica spectrum disorder. We present a patient who developed anti-MOG antibody-positive ADEM following infectious mononucleosis (IM) due to Epstein–Barr virus (EBV) infection.

**Case presentation:**

A 36-year-old healthy man developed paresthesia of bilateral lower extremities and urinary retention 8 days after the onset of IM due to primary EBV infection. The MRI revealed the lesions in the cervical spinal cord, the conus medullaris, and the internal capsule. An examination of the cerebrospinal fluid revealed pleocytosis. Cell-based immunoassays revealed positivity for anti-MOG antibody with a titer of 1:1024 and negativity for anti-aquaporin-4 antibody. His symptoms quickly improved after steroid pulse therapy followed by oral betamethasone. Anti-MOG antibody titer at the 6-month follow-up was negative.

**Conclusions:**

This case suggests that primary EBV infection would trigger anti-MOG antibody-positive ADEM. Adult ADEM patients can be positive for anti-MOG antibody, the titers of which correlate well with the neurological symptoms.

## Background

Myelin–oligodendrocyte glycoprotein (MOG) is exclusively expressed on the surface of oligodendrocytes in the central nervous system (CNS). Anti-MOG antibody is predominantly detected in pediatric acute disseminated encephalomyelitis (ADEM), recurrent optic neuritis, and aquaporin-4 antibody-seronegative neuromyelitis optica spectrum disorder (NMOSD). Recent studies proposed that anti-MOG antibody-associated demyelinating diseases were indeed a clinical spectrum in pediatric patients and that their clinical features were different from those of multiple sclerosis and NMOSD with anti-aquaporin-4 (AQP4) antibody [1, 2]. ADEM is a heterogeneous syndrome that is occasionally triggered by an antecedent infection [3]. A patient with anti-MOG antibody-positive longitudinally extensive transverse myelitis (LTEM) that developed after infection with influenza virus was previously reported [4]. However, no anti-MOG antibody-positive ADEM cases with a preceding viral infection other than influenza have been reported till date. Here we present a patient who developed anti-MOG antibody-positive ADEM following infectious mononucleosis (IM) due to primary Epstein–Barr virus (EBV) infection.

## Case presentation

**Fig. 1:**
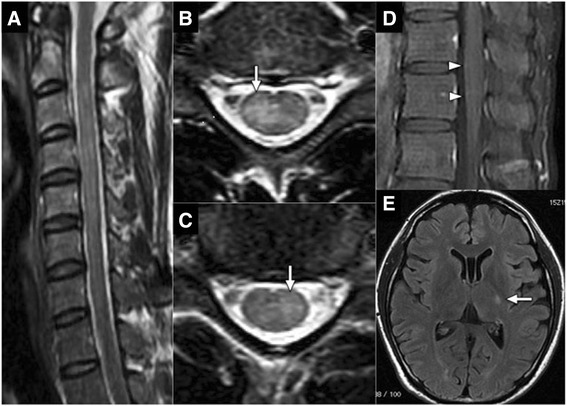
Spinal cord T2-weighted MRI shows a hyperintense lesion extending from C2 to C6 on the sagittal view (**a**), and predominantly in the central gray matter on the axial view at C4 (**b**. *arrow*) and C6 level (**c**. *arrow*). Gadolinium-enhanced T1-weighted MRI shows slight gadolinium enhancement within the conus medullaris surface (**d**. *arrow heads*). Brain fluid-attenuated inversion recovery MRI shows a hyperintense lesion in the left crus posterius capsulae internae (**e**. *arrow*)

A 36-year-old healthy man developed fever and right cervical lymphadenopathy. Laboratory analysis showed elevated white blood count (10,390/mm^3^ with 33% neutrophil, 51% lymphocyte, and 12% atypical lymphocytes), elevated liver enzymes (aspartate transaminase, 193 U/l; alanine transaminase, 413 U/l). Serological studies indicated primary EBV infection (EBV viral capsid antigen [VCA] IgM, positive at 1:40; EBV VCA IgG, positive at 1:160, EBV nuclear antigen IgG, negative). Serologic testing for human immunodeficiency virus antibody was negative. Based on these clinical features, the patient was diagnosed with IM due to primary EBV infection. However, 8 days after onset, the patient developed paresthesia of bilateral lower extremities and urinary retention, which were exacerbated over the next few days. The patient was alert and oriented but had a high fever of 38.5 °C. Neurological examination revealed normal cranial nerves and no weakness in limbs; however, unstable gait with hyperreflexia, sensory disturbance in the entire area below the T7 level, and dysuria that required urethral catheterization were present. Laboratory analysis showed normal white blood count and decreasing liver enzyme levels. Antinuclear and SS-A antibody levels were within normal limits. Cerebrospinal fluid (CSF) examination showed pleocytosis (76/mm^3^), protein concentration of 104.3 mg/dl, IgG index of 0.61, the absence of oligoclonal IgG bands. In addition, IgG and IgM antibodies to EBV VCA and polymerase chain reaction for EBV DNA were negative in the CSF. These findings excluded the direct presence of EBV in the CNS. Additionally, polymerase chain reaction for herpes simplex virus 1, herpes simplex virus 2, and varicella-zoster virus DNA were negative in the CSF. IgG and IgM antibodies to cytomegalovirus were negative in the CSF. These findings excluded viral myelitis. Spinal MRI showed a T2-hyperintense lesion predominantly in the central gray matter extending from C2 to C6 (Fig. 1). Brain MRI showed a fluid-attenuated inversion recovery-hyperintense lesion in the left posterior limb of the internal capsule (Fig. 1). Nerve conduction studies of the left upper and lower extremities showed normal motor and sensory function. Cell-based immunoassays revealed positivity for anti-MOG antibody with a titer of 1:1024 and negativity for anti-AQP4 antibody [2]. Therefore, the patient was started on immunosuppressive therapy with intravenous methylprednisolone (IVMP) for 3 consecutive days, followed by oral betamethasone (2 mg/day). The gadolinium-enhanced spinal MRI after the start of therapy revealed slight gadolinium enhancement of the conus medullaris surface (Fig. 1). However, shortly after IVMP initiation, his symptoms demonstrated significant improvement, and urethral catheter was removed 9 days after the start of IVMP. His sensory disturbance and gait instability was completely resolved 2 weeks after IVMP initiation. Oral betamethasone was tapered following IVMP, and he was discharged without any symptoms or sequelae. Follow-up MRI 1 month after IVMP showed reduction in all CNS lesions. Anti-MOG antibody titer at the 6-month follow-up was negative. No symptomatic recurrence was observed during follow-up evaluation at 11 months after onset. Clinical course, the CSF and MRI findings, and the response to immunosuppressive therapy were most consistent with the diagnosis of anti-MOG antibody-positive ADEM [3, 5].

## Discussion and Conclusions

We present a case of a patient who developed anti-MOG antibody-positive ADEM following IM. In our patient, ADEM occurred relatively early i.e., 8 days after IM onset. However, the absence of EBV genome in the CSF samples is strong evidence for an autoimmune pathogenesis of neurological signs following IM. The present case illustrates two important clinical issues. First, adult ADEM patients can be positive for anti-MOG antibody, the titers of which correlated well with neurological symptoms. Among pediatric ADEM cases, pleocytosis, spinal cord lesions characterized by LTEM, and better outcomes were observed more frequently in patients with anti-MOG antibody than in those with negative titers. Anti-MOG antibody titers of monophasic ADEM patients declined or became negative over the course of months to years [6]. However, patients with persistently high anti-MOG antibody titers experienced a recurrent disease course [6, 7]. The anti-MOG antibody titer of the present case became negative and did not show recurrence. Thus, assessment for anti-MOG antibody titers in adult ADEM patients might be useful in predicting prognosis and determining the course of disease management.

Second, primary EBV infection triggers anti-MOG antibody-positive ADEM. Antecedent infections were reported in 46% of adult ADEM patients [3]. However, those were usually nonspecific upper respiratory tract infections, and systemic viral infections preceding ADEM were rarely reported in adult patients [3]. While several studies previously reported ADEM and LTEM cases associated with EBV infection [8–14], anti-MOG antibody titers were not examined in any of the studies. Recent reports proposed the presence of cross-reactivity between EBV and myelin proteins [15] and between MOG and EBV nuclear antigen [16]. Anti-MOG antibody was detected in 20% of patients with IM due to primary EBV infection without neurological manifestations [17]. These findings implicate EBV infection as a potential trigger for anti-MOG antibody production. However, a potential specific molecular mimicry between antibodies produced in response to EBV antigens and MOG remains unclear. The incidence of neurological involvement in IM was reported to range be 0.37–7.3% [8], and LTEM and ADEM remain very rare complications of EBV infection [8–10]. Therefore, we propose that anti-MOG antibody production might result from synergistic effects of additional unknown factors in response to EBV infection.

In conclusion, this case highlights the possibility that primary EBV infection triggers anti-MOG antibody-positive ADEM. Future studies are necessary to confirm the role of EBV in the pathogenesis of anti-MOG antibody-associated demyelinating diseases.

## Abbreviations

ADEM: Acute disseminated encephalomyelitis
AQP4: Aquaporin-4
CNS: Central nervous system
CSF: Cerebrospinal fluid
DNA: Deoxyribonucleic acid
EBV: Epstein-Barr virus
IM: Infectious mononucleosis
IVMP: Intravenous methylprednisolone
LTEM: Longitudinally extensive transverse myelitis
MOG: Myelin oligodendrocyte glycoprotein
MRI: Magnetic resonance imaging
NMOSD: Neuromyelitis optica spectrum disorder
VCA: Viral capsid antigen
